# Transcription factor Hap2p regulates antioxidant stress responses to maintain miconazole resistance in *Candida albicans*

**DOI:** 10.1080/21501203.2024.2432424

**Published:** 2025-01-06

**Authors:** Yulin Qin, Quanzhen Lv, Hongtao Xu, Yongbing Cao, Bing Han

**Affiliations:** a Institute of Vascular Disease, Shanghai TCM-Integrated Hospital, Shanghai University of Traditional Chinese Medicine, Shanghai, China; b Department of Pharmacy, Minhang Hospital, Fudan University, Shanghai, China; c School of Pharmacy, Naval Medical University, Shanghai, China

**Keywords:** *Candida albicans*, azole susceptibility, Hap2p, anti-oxidative stress, mitochondrial function

## Abstract

Acquired resistance in *Candida albicans* brings about a serious challenge to the clinical application of azoles, so it is urgent to elucidate the mechanisms of azole resistance to improve the therapeutic efficiency. In the aim of searching for the potential targets mediating fluconazole resistance, we screened a mutant library of 48 transcription factor deletion *Candida albicans* strains. The screening results showed that *hap2Δ/Δ* mutants were significantly more susceptible to azoles, especially to miconazole (MCZ). Under MCZ treatment, the intracellular reactive oxygen species (ROS) were significantly higher in *hap2Δ/Δ* mutants compared to the control strain SN250. The addition of antioxidants reversed the MCZ-sensitive phenotype caused by the deletion of *HAP2*. Consistently, the expression of antioxidases responsible for scavenging ROS was shown to decrease in *hap2Δ/Δ* mutants, suggesting that the transcription factor Hap2p is involved in the regulation of oxidative stress responses in *C. albicans*. In addition, *HAP2* deficiency also resulted in impaired mitochondrial function and affected cellular energy supply, which may be related to the iron deficiency regulated by HAP complex. *HAP2* disruption also decreased efflux-mediated resistance of *C. albicans*, as demonstrated by a significant decrease in Cdr1p expression and a slight decrease in Mdr1p expression in *hap2Δ/Δ* strains under the action of MCZ. The above results indicate that the transcription factor Hap2p was required for the resistance of *C. albicans* to azoles, which could provide a new strategy to solve the clinical azoles resistance.

## GRAPHICAL ABSTRACT

**Figure:**
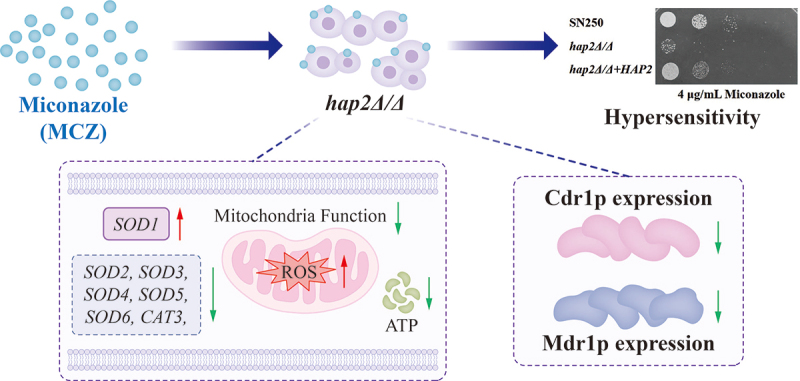

## 1. Introduction

*Candida albicans* is the major opportunistic fungal pathogen that causes superficial infections as well as candidemia and deep-tissue infections in immunocompromised individuals (Lionakis et al. 2023; Wang et al. 2024). Systemic infection caused by *Candida* species, mainly caused by *C. albicans*, is the fourth leading cause of nosocomial bloodstream infection (Lionakis 2014; Liu et al. 2014; Ramirez-Zavala et al. 2014). The increasing rate of non-*albicans* isolation and the rapidly growing resistance of *Candida* species to azoles present a challenging problem in clinical settings (Berkow and Lockhart 2017). About 7% of all *Candida* bloodstream isolates tested were resistant to fluconazole from the statistics of Center for Disease Control (CDC) in the US due to the general and long-term use (Husni et al. 2023). The incidence of azole resistance was up to 5% in *C. albicans* isolates from candidemic patients (Whaley et al. 2016). The emergence of resistance to the presently limited antifungal drugs seriously impedes the effective treatment of invasive infections. The estimate of mortality from *Candida* infections was as high as 45% (Pristov and Ghannoum 2019). Therefore, a thorough understanding of the mechanistic principles of antifungal drug resistance is essential for developing novel strategies to treat fungal infections.

Currently, azole resistance is mediated by three main mechanisms: drug target alteration (Erg11p), drug target overexpression (Erg11p), and efflux pumps overexpression (Cdr1p, Cdr2p, Mdr1p) (Lee et al. 2021). Many details of the molecular basis of drug resistance in *C. albicans* have been elucidated in recent years, especially with the identification of transcription factors that regulate the expression of ergosterol biosynthesis genes and drug transporters (Berkow and Lockhart 2017). There are 258 transcription factors in *C. albicans* of which 6 classical ones have been identified as being involved in the regulation of antifungal drug resistance, including Upc1p, Mrr1p, Cap1p, Tac1p, Cas5p, and Ndt80p (MacPherson et al. 2005; Sasse et al. 2011; Xie et al. 2017; Feng et al. 2018; Liu et al. 2020). Many recent studies have shown that multiple transcription factors are involved in the regulation of azole susceptibility through the regulation of fungal ergosterol pathway, virulence, efflux pump expression, etc., suggesting that transcription factors are a potential repertoire for the discovery of antifungal drug targets (Shrivastava et al. 2023).

In this study, a library of 48 transcription factor-deficient strains of *C. albicans* was screened for susceptibility to azoles with the aim of identifying new transcription factors with drug susceptibility modulation. Screening results showed that *HAP2* deficiency causes increased susceptibility to azoles, especially to miconazole. Our research found that Hap2p could regulate fungal cell oxidative responses to miconazole and maintain the expression of efflux pumps, mediating azoles resistance in *C. albicans*.

## 2. Materials and methods

### 2.1. Strains, media, and agents

All the strains and primers used in this study are listed under supplemental Table S1 and Table S2 respectively. The *C. albicans* strain SN152 and 48 transcription factor deletion *C. albicans* mutants are gifts from Suzanne Noble and A. D. Johnson. SN250 was used as the control strain for all strains were constructed on the basis of SN152 and introduced the *C. dubliniensis HIS1* and *C. maltosa LEU2* makers. *Candida albicans* strains were routinely grown in yeast peptone dextrose medium (YPD, 2% dextrose, 2% peptone, 1% yeast extract) at 30 °C. Plates were prepared with 2% agar for all solid media. Antifungal agents used in this study (fluconazole, miconazole, itraconazole, voriconazole, ketoconazole, clotrimazole, bifonazole, terbinafine, 5-fluorouracil, and caspofungin) were purchased from Selleck. DMSO was purchased from Sinopharm Chemical Reagent, Co., Ltd. Drug stock solutions were prepared using dimethyl sulphoxide (DMSO) as a solvent and stored at −20 °C. For iron-dependent assays, YPD medium was defined as high-iron medium and YPD plus 100 μmol/L or 200 μmol/L bathophenanthroline disulfonic acid (BPS, Sigma, the iron chelator) was defined as low-iron medium. Hydroxyphenyl fluorescein (HPF) was provided by Maokang Biotechnology Co., Ltd.

### 2.2. Strain construction

The information of *HAP2* reintroduction strains and the primers used in strain construction are listed in supplementary Table S1 and Table S2 respectively. *HAP2* complemented strains were constructed by the plasmid pRS316 through homologous recombination method. Fusion PCR was used to amplify the upstream and downstream fragments of *C. maltose LEU2*, the cassette of *HAP2* and its upstream 1,500 bp and downstream 500 bp, as well as *C. dubliniensis ARG4*. All four DNA fragments and plasmid pRS316 after *Bam*H I digestion were transferred into *Saccharomyces cerevisiae* strain W303 to generate recombinant plasmids and homologous recombinant fragments after *Eco*R I digestion. The recombinant *HAP2* strains were identified by selection for the *ARG4* auxotrophic marker.

### 2.3. Drug susceptibility testing

#### 2.3.1. Minimum inhibitory concentration (MIC) assay

Minimum inhibitory concentration (MIC) assay was determined according to the broth microdilution method detailed by the Clinical and Laboratory Standards Institute (CLSI) in documents M27-A3 (Lv et al. 2018). *C. albicans* were incubated in YPD overnight at 30 °C and harvested after three washes with PBS. Cells were harvested and diluted with RPMI1640 at a concentration of 5 × 10^3^ CFU/mL. Fungal cell suspension was added to 96-well plates at 100 μL each well except blank control. Antifungals were added from the second column and diluted two-fold serially, followed by incubation at 30 °C. OD_630_ was measured at 24 h and 48 h respectively using a microplate reader. The lowest concentration of the antifungals that showed prominent inhibition of growth (approximately 80%) was defined as the MIC for fluconazole. For miconazole, a growth inhibition of more than 99% was defined as MIC.

#### 2.3.2. Spot assay

Spot assay was performed to determine the drug susceptibility of strains on YPD agar plates. *C. albicans* cells were cultured in YPD overnight for 16 h to reach the mid-log phase. Cells were harvested and diluted to 2 × 10^6^ cells/mL and then 10-fold diluted in sterile PBS successively. Five concentration gradients of fungal cells were spotted on YPD plates containing different concentrations of drugs. Images of the plates were taken after 48 h of incubation.

#### 2.3.3. Growth curve experiment

*Candida albicans* cells were cultured in YPD overnight for 16 h to reach the mid-log phase. *C. albicans* cells were propagated in YPD broth and final cell concentrations were adjusted to an initial optical density at OD_630_ of 0.1. All cells were incubated in YPD media with the corresponding dosing solution at 30 °C with 200 r/min shaking. Growth curves were plotted using the OD_630_ values measured during the culturing period.

### 2.4. Intracellular ATP levels measurement

Intracellular ATP levels were measured using the BacTiter-Glo^TM^ Microbial Cell Viability (Promega, USA) according to the manufacturer’s instructions. Briefly, *C. albicans* strains were cultured in YPD overnight and harvested at exponential time. A total of 2 × 10^6^ cells were concentrated in 100 μL YPD as samples and 100 μL BacTiter-Glo reagent was added. Luminescence was recorded by Tecan Infinite M200 microplate reader.

### 2.5. Determination of mitochondrial membrane potential

The fluorescent dye JC-1 (Beyotime Biotechnology, China) was used to measure cell mitochondria (Δψm) according to the manufacturer’s instructions. *C. albicans* strains were cultured in YPD overnight and harvested at exponential time and sample cells were adjusted to 2 × 10^7^ CFU/mL. A mixture of 500 μL JC-1 staining working solution and 500 μL sample cell culture was incubated at 30 °C, 200 r/min for 30 min. The sample cell cultures were then harvested by centrifugation and washed twice with JC-1 buffer. The cell pellets were resuspended with 100 μL JC-1 buffer. Fluorescence intensity was measured at an excitation wavelength of 490 nm, emission wavelength of 530 nm and excitation wavelength of 490 nm, emission wavelength of 590 nm, respectively using a Tecan Infinite M200 microplate reader in a dark 96-well plate. The mitochondrial membrane potential is proportional to the ratio of the measured red fluorescence to the green fluorescence intensity.

### 2.6. Rhodamine 6 G (Rh6G) efflux assay

Exponentially growing *C. albicans* cells were harvested and washed three times in PBS. Cell pellets were resuspended in PBS and incubated for 2 h at 30 °C at 200 r/min for energy dissipation. Rh6G was added to the above samples and the sample cells were incubated at a final concentration of 10 μmol/L, 30 °C, 200 r/min for 1 h to allow Rh6G accumulating to fungal cells. After incubation, sample cells were washed with PBS thoroughly and the final cell concentration was adjusted to 5 × 10^7^ CFU/mL exactly. D-glucose was added to the final concentration of 2 mmol/L to initiate Rh6G efflux. The fluorescence intensity of each sample was measured at 0, 15, 30, 45, 60, and 90 min to determine the amount of Rh6G efflux at an excitation wavelength of 515 nm and an emission wavelength of 555 nm.

### 2.7. Relative quantification of mRNA by real-time RT-PCR

*Candida albicans* cells were grown overnight and harvested at exponential time by centrifugation. Total RNA samples from *C. albicans* strains were extracted using the Column Fungal RNAout Kit (KangLang, China) and the RNA sample concentration was measured using the Nanodrop spectrophotometer. Total RNA was treated with genomic DNA Eraser (TaKaRa, China) to remove the residual DNA and reverse transcribed using the PrimeScript RT Reagent Kit (TaKaRa, China). Real-time PCR was performed using the SYBR Green Master Mix (TaKaRa, China) on the Fast Real-Time PCR System (AB Applied Biosystems 7900HT). Primers used to detect the expression of each gene are listed in Table S2.

### 2.8. Differential gene expression analysis

Exponentially growing *C. albicans* cells were harvested and washed three times with PBS. RNA was extracted from the *C. albicans* SN250 and *hap2Δ/Δ* mutants treated with dimethyl sulphoxide (DMSO) or 4 μg/mL of miconazole for 3 h using a column fungal RNAout kit (KangLang, China). The quality of RNA samples was detected based on the A_260_/A_280_ absorbance ratio with a Nanodrop ND-2000 system (Thermo Scientific, USA). Qualified samples will be used for library construction and the library preparations were sequenced on an Illumina Novaseq 6000 (or MGISEQ-T7). Differential expression analysis was performed using the DESeq2, DEGs with |log2FC| > 1 and *P*.adj < 0.05 were considered to be significantly different expressed genes. Go function enrichment and KEGG pathway enrichment analysis were performed by cluster Profiler R software package. GO or KEGG function is significantly enriched when *p* < 0.05.

### 2.9. Intracellular ROS production assay

Hydroxyphenyl fluorescein (HPF) (Maokang, China) was used for detecting intracellular ROS production and the detection method was described previously (Wang et al. 2023). *C. albicans* cells were cultured overnight for 16 h and harvested at exponential time. After washing and resuspension, *C. albicans* cells were diluted 1:100 in YPD and treated or untreated with miconazole (8 µg/mL, 12 µg/mL) for 0, 3 h at 200 r/min, 30 °C. After treatment, sample cells were harvested and washed PBS thoroughly. HPF was added to the final cell concentration of 5 µmol/L in PBS. After incubation at 200 r/min, 37 °C for 40 min, all the sample cells were washed with PBS and finally analysed by microplate reader.

## 3. Results

### 3.1. *The deletion of* HAP2 *increased sensitivity of* C. albicans *to azoles, especially to miconazole*

**Figure 1.**
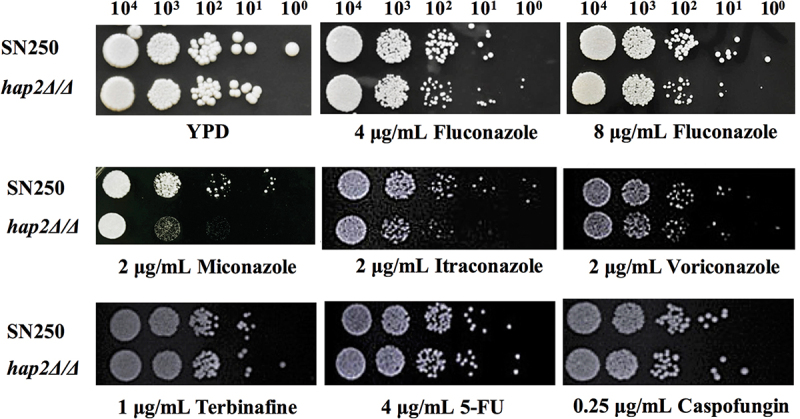
The *hap2Δ/Δ* strain displayed enhanced susceptibility to azoles. Exponentially growing strains (SN250 and *hap2Δ/Δ*) were spotted in serial dilutions onto YPD plates containing different concentrations of antifungal agents. Abbreviation: FLC, fluconazole; MCZ, miconazole; ICZ, itraconazole; VCZ, voriconazole; 5-FU, 5-fluorouracil; CAS, caspofungin.

**Figure 2.**
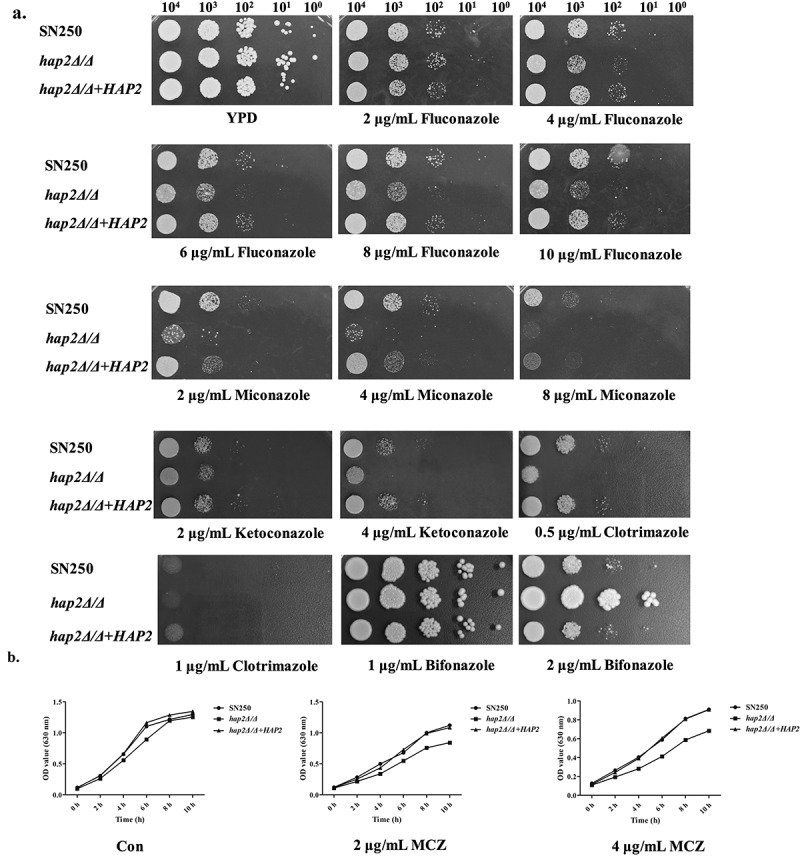
The *hap2Δ/Δ* strain displayed enhanced susceptibility to miconazole. (a) Exponentially growing strains (SN250, *hap2Δ/Δ*, and *hap2Δ/Δ+HAP2*) were spotted in serial dilutions onto YPD plates containing different concentrations of FLC and MCZ. (b) Growth curves of strains (SN250, *hap2Δ/Δ*, and *hap2Δ/Δ+HAP2*) in YPD under the effects of miconazole (2 μg/mL, 4 μg/mL) at 30 °C. All the test strain cells from 30 °C, 200 r/min overnight cultures were harvested, washed, and subcultured (initial OD_630_ = 0.1) in liquid YPD medium. The cultures were subsequently incubated at 30 °C for indicated time.

To identify the transcription factors involved in the regulation of fungal sensitivity to azoles, we screened a library consisting of 48 transcription factor-deficient *C. albicans* strains. These transcription factors have a wide range of functions and are involved in the regulation of drug resistance, virulence, biosynthesis, stress response etc (Table S3). The screening results showed that *HAP2* deletion significantly enhanced the susceptibility of *C. albicans* to fluconazole and miconazole, which had the minimal MIC of FLC and MCZ among all the 48 mutants. According to the MIC value of FLC for 24 h, we classified the 48 mutants into three categories: (1) “sensitive strains” (0 μg/mL < MIC < 4 μg/mL); (2) “moderately resistant strains” (4 μg/mL ≤ MIC < 16 μg/mL); (3) “resistant strains” (16 μg/mL ≤ MIC ≤ 64 μg/mL) (Table S3). There are 6 out of 48 strains that belong to the “sensitive strains”. Of the 48 mutants, *HAP2*-deficient strains showed the most significant increase in susceptibility to FLC and MCZ (Figure S3). Furthermore, antifungal susceptibility of *hap2Δ/Δ* mutants was confirmed by MIC (Table 1), spot assays, and growth curve assays (Figure 1). In consistent with the screening results, the *hap2Δ/Δ* mutants exhibited increased susceptibility to azoles including fluconazole, miconazole, itraconazole, and voriconazole, especially sensitive to miconazole (Figure 1). We also tested other imidazoles, such as ketoconazole, clotrimazole, and bifonazole. We found that *hap2Δ/Δ* mutants were more sensitive to ketoconazole and clotrimazole compared to fluconazole, but were resistant to bifonazole (Figure 2). By contrast, *HAP2* deletion didn’t cause the susceptibility alteration of *C. albicans* to other kinds of antifungal drugs such as caspofungin, terbinafine, and 5-fluorouracil. To confirm the specificity of *HAP2* in modulating azole resistance, we constructed the *HAP2* revertant strain. By detecting MIC and spot assay, we found that *HAP2* supplementation fully recovered *C. albicans* resistance to azoles. Overall, our studies revealed 6 transcription factors regulating the susceptibility of *C. albicans* to azoles drugs, among which *hap2Δ/Δ* mutant showed the highest sensitivity.

**Table 1.** MIC (μg/mL) values of *HAP2* deficient *Candida albicans* isolates to fluconazole and miconazole.

Strains	MIC (μg/mL)		MIC (μg/mL)	
	FLC (24 h)	MCZ (24 h)	FLC (48 h)	MCZ (48 h)
SN250	>64	16	>64	16
*hap2Δ/Δ*	0.0625	0.0625	0.25	0.0625
*hap2Δ/Δ+HAP2*	>64	16	>64	16

FLC: Fluconazole; MCZ: Miconazole.

### 3.2. *The miconazole sensitive phenotype of* HAP2-*deficient strains was associated with decreased antioxidant capability*

**Figure 3.**
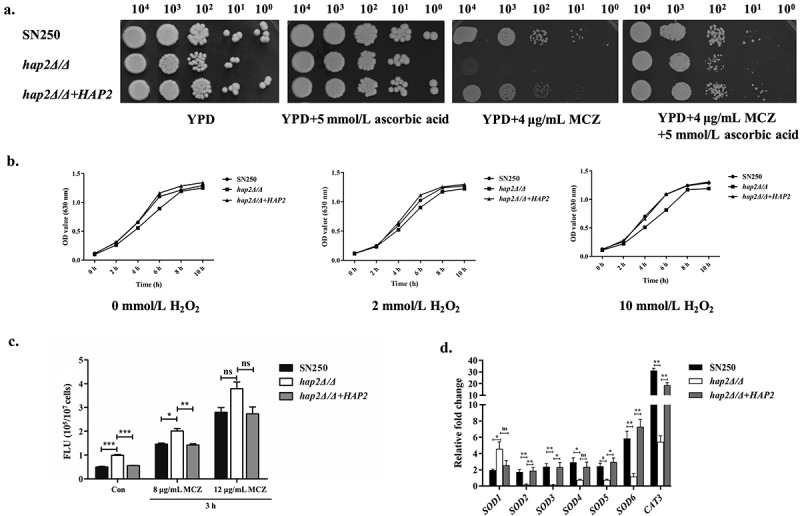
The influence of Hap2p on the anti-oxidative responses. (a) Spot assay was performed to determine the miconazole susceptibility changes of *Candida albicans* under the treatment of ascorbic acid. Exponentially growing strains were spotted in serial dilutions onto YPD plates containing different concentrations of antifungal agents (miconazole: 4 μg/mL, ascorbic acid: 5 mmol/L) for 48 h. (b) Growth curves of strains in YPD under the effects of H_2_O_2_ (0 mmol/L, 2 mmol/L, 10 mmol/L) at 30 °C. All the test strain cells were incubated in YPD or YPD with different concentrations of H_2_O_2_ for 24 h at 30 °C with 200 r/min shaking (initial OD_630_ = 0.1) and OD_630_ values were measured. (c) Intracellular ROS production of *C. albicans* cells was measured under different conditions (8 μg/mL or 12 μg/mL miconazole for 3 h). About 1 × 10^7^ cells in exponential growth phase were collected, washed with PBS, stained with 5 mmol/L of HPF, and analyzed by microplate reader. FLU: Fluorescence. (d) Fold changes in the expression levels of antioxidant genes (*SOD1-6*) in *hap2Δ/Δ* compared to SN250. Each sample was processed in triplicate. Error bars show the standard deviation of the mean. Data are shown as mean ± standard deviation and analysed by one-way ANOVA (c) and unpaired t test (d), ns: Not significant; *p < 0.05; **p < 0.01; ***p < 0.001.

Since *HAP2*-deficient isolates showed specific hypersensitivity to miconazole among all the azoles, we speculated that transcription factor Hap2p might be involved in regulating the oxidative stress responses against the damage of miconazole. As previously reported, in addition to inhibiting ergosterol biosynthesis in fungi as the common mechanism of azoles, miconazole can also inhibit the activity of peroxidase and exert fungicidal effects by causing oxidative stress (Snell et al. 2012; Tits et al. 2021). While other azoles do not cause robust oxidative stress in fungal cells. Our Spot assay results revealed that ascorbic acid, a reducing agent, significantly reversed the miconazole-sensitive phenotype caused by *HAP2* deletion (Figure 3a). At the second diluted spot, the growth of *HAP2*-deficient mutants was basically consistent with that of the control and revertant strains in the presence of MCZ and ascorbic acid. However, at the third and fourth diluted spots, the growth of *HAP2*-deficient mutants was still slightly lower than that of the control and recovery strains, which may be due to the impaired mitochondria and reduced efflux function mentioned in our follow-up results. Meanwhile, the same dose of H_2_O_2_ more significantly inhibited the growth of *hap2Δ/Δ* mutant, indicating that the *HAP2*-deficient *C. albicans* were more sensitive to oxidative stress (Figure 3b). To measure the amount of intracellular ROS production, we conducted a fluorometric assay using hydroxyphenyl fluorescein (HPF; 5 μmol/L). We found that there was an obvious intracellular ROS increase in the mutants compared to SN250 (Figure 3c). Further measurement of representative antioxidant gene (*SOD1-6* and *CAT3*) transcript levels was performed by qRT-PCR analysis in the mutants. The results showed that *SOD2-6* genes and *CAT3* gene expression were significantly downregulated in *hap2Δ/Δ* mutants under the effects of miconazole. However, qRT-PCR analysis showed inconsistent expression levels of *SOD1* and *SOD2-6*, which may be due to the fact that *SOD1* expression is not regulated by Hap2p, whereas it’s the high levels of intracellular ROS that induce the upregulation of *SOD1* expression (Figure 3d) (Haque et al. 2019).

**Figure 4.**
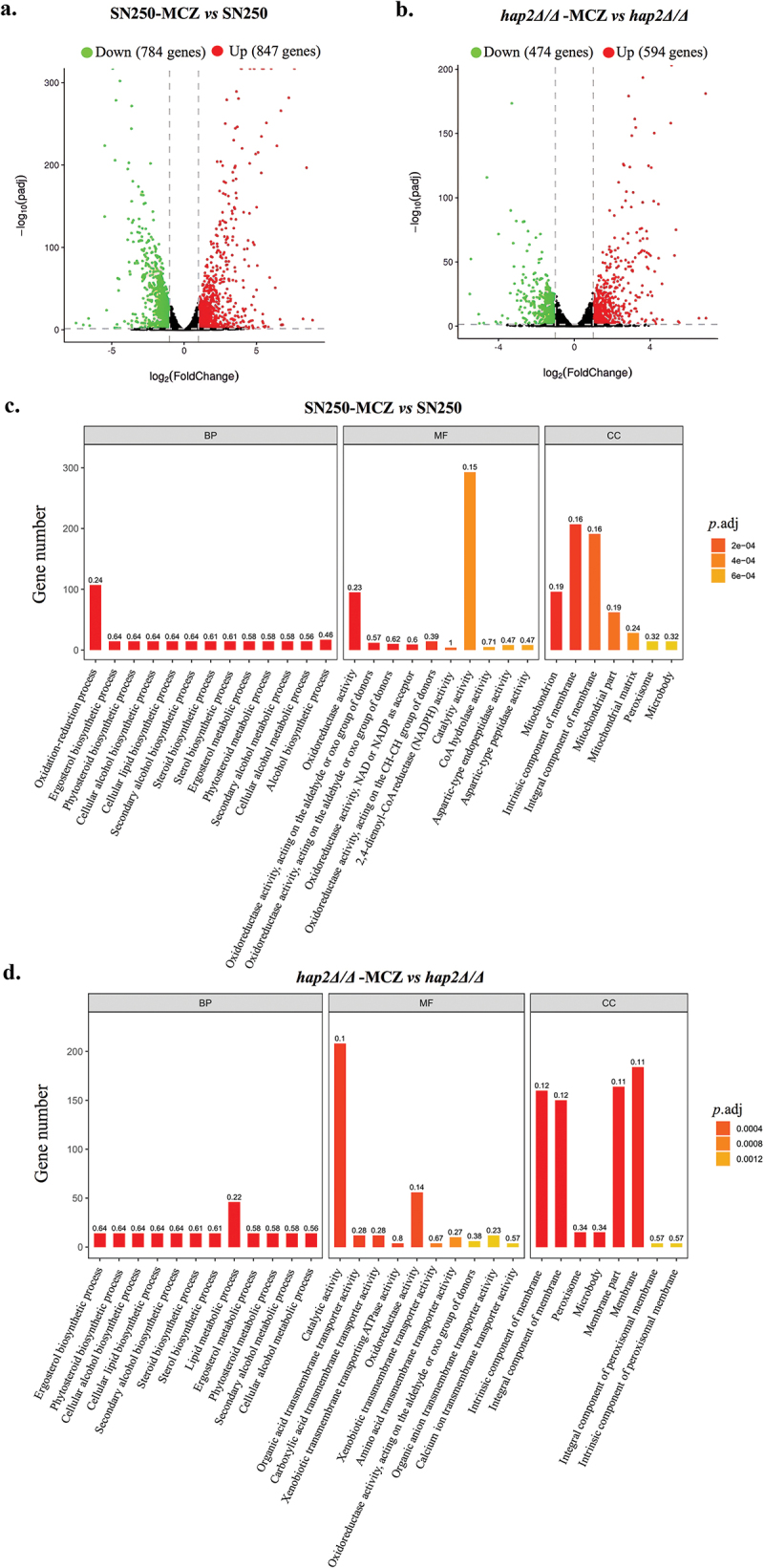
Differential gene expression analysis. Differential gene expression analysis on the effects 4 μg/mL for 3 h. *Candida albicans* strains: SN250, *hap2Δ/Δ*. Volcano plot of *C. albicans* genes showing altered in the presence *vs* absence miconazole in (a) SN250 and (b) *hap2Δ/Δ*. The red points represented genes that were significantly upregulated in each group; the green points represented genes that were upregulated significantly in each group (adjusted *p* < 0.05). Gene ontology (GO) enrichment analysis in the presence *vs* absence miconazole in (c) SN250 and (d) *hap2Δ/Δ*. BP: Biological process; MF: Molecular function; CC: Cellular component; *P*.adj: Adjusted *p* value.

To comprehensively investigate the effect of *HAP2* on the gene’s expression, we determined the transcriptome of *C. albicans* treated with miconazole. The volcano plot indicated that a total of 1,631 DEGs (Differentially Expressed Genes) were identified in SN250 strain under the treatment of miconazole (4 μg/mL, 3 h), whereas 1,068 DEGs were present in *hap2Δ/Δ*. In comparison, there were fewer up-regulated genes in *hap2Δ/Δ* mutant (594 genes) than in SN250 strain (847 genes) (Figure 4a,b). The details of the transcriptome analysis, including gene ID, gene description, fold changes, and statistical significance, are provided in Table S4 (SN250-MCZ *vs* SN250) and Table S5 (*hap2Δ/Δ*-MCZ *vs hap2Δ/Δ*). GO enrichment analysis was performed to gain further insights into functional implications of these differentially expressed genes. The results of GO enrichment analysis showed that MCZ could upregulate the expression of multiple oxidative stress-related genes in SN250 (Figure 4c). However, there was no significant enrichment of oxidative stress genes in *HAP2* deficient strains, suggesting that Hap2p may affect the transcription of oxidative stress-related genes (Figure 4d). The details of the GO enrichment analysis data were provided in Table S6 (SN250-MCZ *vs* SN250) and Table S7 (*hap2Δ/Δ*-MCZ *vs hap2Δ/Δ*). All these results demonstrated that the destruction of *HAP2* resulted in a decreased antioxidant stress ability of *C. albicans*, thus increasing its sensitivity to miconazole.

### 3.3. *The disruption of* HAP2 *resulted in impaired mitochondrial function and reduced efflux*

**Figure 5.**
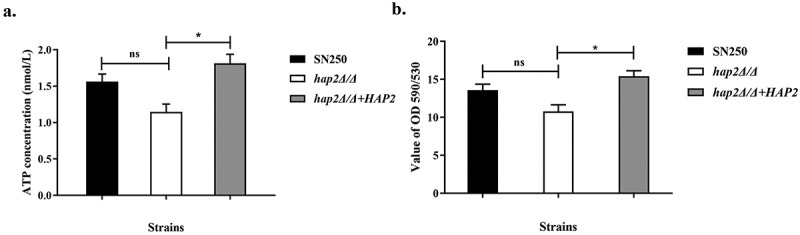
Deletion of Hap2p resulted in impaired fungal mitochondrial respiratory function. (a) Effect of *HAP2* loss on intracellular ATP levels. Different strain cells were collected and intracellular ATP level was determined. (b) Mitochondrial membrane potential Δψm of SN250, *hap2Δ/Δ* and *hap2Δ/Δ+HAP2*. Data are shown as the mean ± SD of three independent experiments and analysed by unpaired t test (a, b), *, *p* < 0.05; ns, not significant.

**Figure 6.**
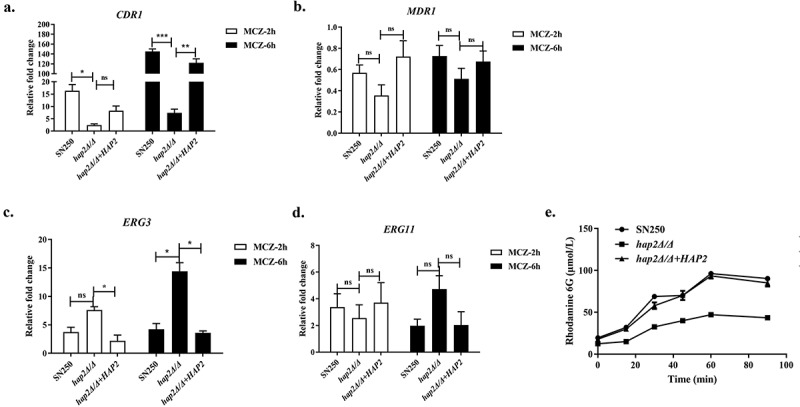
Hap2p mediated drug resistance in *Candida albicans* by regulating the activity of efflux pump. (a–d) RT-PCR analysis of *CDR1*, *MDR1*, *ERG3*, and *ERG11* expression in different strains (SN250, *hap2Δ/Δ*, and *hap2Δ/Δ*+*HAP2*) expressed as fold-change under the treatment of MCZ for 2 h and 6 h. (e) The ability of *C*. *albicans* strains (SN250, *hap2Δ/Δ*, and *hap2Δ/Δ*+*HAP2*) to pump out Rh6G in the presence of glucose. Measurement of supernatant fluorescence reflects the ability of the efflux pump. Data are shown as the mean ± SD of three independent experiments and analysed by one-way ANOVA (A-D), *p < 0.05; **p < 0.01; ***p < 0.001; ns, not significant.

Mitochondria is recognised as the powerhouse of eukaryotic cells, which produce ATP for cell energy supply. Considering that reduced cellular resistance to oxidative stress may be highly related to abnormal mitochondrial functions, which also affect sensitivity to miconazole, we measured the intracellular ATP levels and mitochondrial membrane potential of *hap2Δ/Δ* mutant. Our results showed that the deletion of *HAP2* caused both intracellular ATP levels and mitochondrial membrane potential (Δψm) to decrease, indicating the likelihood of impaired mitochondrial function and mitochondrial aerobic respiratory metabolism dysfunction (Figure 5a,b). Mitochondrial respiration provides energy for efflux pumps which is one of the important factors in mediating drug resistance to azoles in fungi. To verify whether the expression of drug resistance genes is regulated by transcription factor Hap2p, qRT-PCR analysis was performed to measure the expression of azole-targeting enzyme genes and efflux pump genes. As shown in Figure 6a, the expression of *CDR1* was significantly upregulated in *C. albicans* treated with MCZ, however, it was not expressed highly in *hap2Δ/Δ* mutant. Compared with the control strain SN250 and revertant *hap2Δ/Δ*+*HAP2* mutant, *CDR1* expression was decreased by nearly 17-fold in *HAP2-*deficient strains under the treatment of MCZ. The presence of the Hap2p transcription factor binding sequence CCAAT in the upstream sequence of the *CDR1* gene suggests that Hap2p may be involved in the regulation of *CDR1* expression. Comparatively, a minor reduction in *MDR1* expression levels occurred in *HAP2*-deficient strains (Figure 6b). Meanwhile, we evaluated the efflux capability by monitoring the extracellular fluorescence intensity of Rh6G. As shown in Figure 6e, the fluorescence intensity detected outside *hap2Δ/Δ* cells was significantly lower than that of the control strain SN250, which indicated *HAP2* deficiency resulted in decreased efflux pump viability. In addition, the expression of azole target encoding gene *ERG11* and *ERG3* in *HAP2* mutant under the action of MCZ was higher than that of SN250 (Figure 6d). *ERG3* is a gene encoding sterol Δ5,6-desaturase, which has been demonstrated to catalyse the synthesis of toxic sterol in *C. albicans* (Hirayama et al. 2020). In our results, *HAP2* deletion resulted in a higher transcription level of *ERG11* and *ERG3*. This high expression may be the negative feedback caused by the higher sensitivity of *C. albicans* to azoles.

## 4. Discussion

As the first-line therapy for fungal infection, azoles have been widely used in clinical treatment for a long time. The development of azole resistance is associated with the long-term and extensive use of azole antifungals, as drug exposure drives the emergence of drug resistance (Lockhart et al. 2023). Azole resistance mechanisms among *Candida* spp. are well characterised, including drug transporters upregulation (Mdr1p, Cdr1p, Cdr2p), drug target alteration or overexpression (*ERG11* mutation) and cellular stress responses (*ERG3* mutations and other stress-response pathways in Hsp90, Sgt1, calcineurin, KDACs, PKC mediated) (Zhang et al. 2020; Lee et al. 2021). As mentioned above, there are several transcription factors involved in the regulation of antifungal susceptibility. Among them, the zinc cluster transcription factor Upc2p is confirmed to regulate genes related to ergosterol biosynthesis including *ERG11* (Hoot et al. 2011; Schubert et al. 2011). The alterations of the Upc2p activity may be responsible for overexpression of *ERG* genes in azole-resistant clinical isolates of *C. albicans* (Schubert et al. 2011). And the gain-of-function mutations in Mrr1p results in the upregulation of efflux pumps encoding gene, *MDR1* (Ramirez-Zavala et al. 2014). The promoter of *MDR1* gene could also be occupied by Cap1p which is involved in the regulation of oxidative stress response (Schubert et al. 2011). Cap1p induces constitutive *MDR1* upregulation upon H_2_O_2_ and increases fluconazole resistance (Mogavero et al. 2011). The overexpression of multidrug transporter gene *CDR1*, *CDR2* is highly related to *C. albicans* azoles resistance. Gain-of-function mutations in Tac1p cause hyperactivity and overexpression of *CDR1* and *CDR2* (Coste et al. 2006). Transcription factor Ndt80p in *C. albicans* also participates in modulating azole tolerance by controlling the expression of *CDR1* (Sellam et al. 2009). Recently, transcription factor Cas5p has been found to govern antifungal drug resistance probably related to its control over cell wall stress and cell circle in *C. albicans* (Xie et al. 2017).

Our preliminary research conducted a screening of transcription factor mutant library for the purpose of searching for new regulatory factors required for the azole resistance. Azoles containing imidazoles (e.g. miconazole, ketoconazole, clotrimazole, and bifonazole) and triazoles (e.g. fluconazole, voriconazole) exert antifungal effects by binding to lanosterol 14α-demethylase (Erg11p). *HAP2*-deficient mutants were more susceptible to miconazole, ketoconazole, clotrimazole, but were resistant to bifonazole. The common antifungal mechanism of imidazoles is inhibition of ergosterol synthesis, but some of them have other unique antifungal pathways. For example, miconazole can kill *C. albicans* cells by inducing intracellular ROS production and bifonazole has been shown to inhibit chitin synthesis. These specific mechanisms may be the important reasons for the different sensitivities of *hap2Δ/Δ* mutant to imidazoles.

Our research showed that the mechanism by which Hap2p regulates azole resistance is closely linked to the high expression of efflux pump and antifungal resistant genes. In *HAP2*-deficient strains, the expression of *CDR1* and *MDR1* could not be up-regulated as much as the control strains under the treatment of MCZ. Also, the efflux pump viability was obviously decreased, which was consistent with the changes in gene expression levels. In addition, *HAP2* deficiency resulted in a higher expression of the azole resistance genes *ERG11* and *ERG3* under MCZ induction. Fungal cells would give negative feedback to upregulate the expression of target genes *ERG11* in response to MCZ treatment. Studies have shown that azole resistance can be due to the loss of function of Erg3p for its role in catalysing the conversion of non-toxic sterol 14α-fecosterol to the toxic sterol 14α-methylergosta-8,24-dien-3β,6α-diol (Luna-Tapia et al. 2018; Hirayama et al. 2020). Erg3p mutation will restrain toxic sterol synthesis pathway and result in the total conversion of lanosterol, the initial substance, to Erg11p (Luna-Tapia et al. 2018; Spettel et al. 2019). However, there is no precise evidence that the transcription factor Hap2p is a direct regulator of the expression of *CDR1*, *MDR1, ERG11*, and *ERG3*. In addition, the expression of *MRR1* was upregulated in the parental SN250 strain but not in the *hap2Δ/Δ* mutant in the RNA-seq, which indicated that the downregulation of *MDR1* may related to *MRR1* (Morschhäuser et al. 2007). In the future, identifying the genes directly regulated by transcription factor Hap2p by CHIP-seq assay will provide more insights.

Hap2p has been reported as a subunit of CCAAT sequence binding factor, HAP complex. HAP complex comprises Hap2p, two distinct homologs of Hap3p (Hap31p, Hap32p), Hap5p, and three putative homologs of Hap4 (Hap41p, Hap42p, and Hap43p) in *C. albicans* (Hsu et al. 2013). Hap2p is substantiated to be involved in the regulation of iron homoeostasis as a subunit of HAP complex which is regulated by Hap43p in *C. albicans* (Singh et al. 2011; Srivastav et al. 2021). Iron homoeostasis is indispensable in invasive fungal cells for its vital role in nutrient metabolism and stress response regulation. Previous studies have proved that iron deficiency enhances drug susceptibility of *C. albicans* by increasing membrane fluidity which results in reduced *ERG11* expression and ergosterol level (Davis et al. 2011; Chen et al. 2024). Transcriptome analysis revealed a correlation between calcineurin signalling and iron homoeostasis, as iron deprivation was associated with downregulation of calcineurin signalling genes, including *HSP90*, *CMP1*, and *CRZ1*, resulting in the susceptibility of strains to stresses such as alkaline pH, salinity and membrane perturbations (Davis et al. 2011). Furthermore, iron deficiency causes intracellular calcium overload and ROS overproduction in *Aspergillus fumigatus* (Ye et al. 2022). Conversely, calcium has inhibition effects on the expression of iron uptake related genes, especially transcription factor HapXp, which indicated a similar mode of modulation in *C. albicans*. Our research showed that *HAP2* deficiency decreased *C. albicans*, increased azole susceptibility and decreased *ERG11* transcript levels, which may be closely related to the *HAP2*-deficient stains poor adaptation to iron deficient environments (Figure S1).

The subunits of the HAP complex, Hap2p, Hap31p/32p, and Hap5p are actively involved in regulating oxidative stress genes (e.g. *CAT1*, *SOD4*, *GRX5*, and *TRX1*) in response to iron availability (Quinn et al. 2017). Our results showed *HAP2* deletion resulted in increased intracellular ROS production in response to MCZ and mitochondrial respiratory dysfunction. Also, qRT-PCR analysis verified that the expression of antioxidant genes (*SOD1-6*) in *HAP2*-deficient strains was lower than that of control strain SN250 in the presence of MCZ. Hap2p may lead to increased ROS production by regulating some of the antioxidant gene expression or mitochondrial oxidative respiration. Since mitochondrial dysfunction in *C. albicans* is known to be associated with antifungal drug susceptibility, cell wall integrity, and virulence, Hap2p is likely to be involved in regulating these functions (Thomas et al. 2013; Hu et al. 2024). Studies have shown that an increase in intracellular ROS contributes to the higher susceptibility of the strain to azoles. Such as the increase in endogenous ROS significantly enhances the synergistic effect of FLC and berberine against FLC-resistant *C. albicans*. Berberine causes mitochondria dysfunction and stimulates the production of ROS (Xu et al. 2009). Another naturally occurring compound, asiatic acid was found to significantly increase intracellular ROS levels when used in combination with FLC compared to FLC or asiatic acid alone (Wang et al. 2021). Miconazole differs from other azoles in that it can inhibit peroxidases and lead to an excessive accumulation of peroxide, which serves as an additional mechanism of action. Consistent with these studies, our results revealed that deficiency of *HAP2* specifically improved miconazole susceptibility and increased intracellular ROS accumulation, which was consistent with the synergistic effects of ROS-increasing drugs with azoles. Elevated intracellular ROS levels are associated with impaired mitochondrial respiratory function, therefore rendering the alterations in the azole susceptibility of fungal cells. The molecular mechanism of iron deficiency and mitochondrial metabolism dysfunction caused by *HAP2* disruption still needs to be further explored. In conclusion, Hap2p has been identified as a key factor in the regulation of oxidative stress responses, mitochondrial function, and azole resistance in *C. albicans*, which could provide a new way to solve the problems of azoles resistance.

## Supplementary Material

Revised Supplementary_Materials.docx
