# Supplementary material for: Transcription factor Hap2p regulates antioxidant stress responses to maintain miconazole resistance in Candida albicans
Source: Mycology. 2025 Jan 6;16(3):1386–99. doi: 10.1080/21501203.2024.2432424 (PMC12422037; doi:10.1080/21501203.2024.2432424)
Supplement: Revised Supplementary_Materials.docx [file TMYC_A_2432424_SM8361.docx]

**Tables S1.** Strains used in this study.

| Strain | Parental strain | Key Genotype | Reference |
| --- | --- | --- | --- |
| SC5314 | Prototroph | Wild-type clinical isolate |  |
| SN152 | SC5314 | *LEU2/leu2 HIS1/his1 arg4/arg4 URA3/ura3△::imm^434^ IRO1/iro1△::imm^434^* | Noble and Johnson 2005 |
| SN250 | SN152 | *leu2△::C.m.LEU2/leu2△::C.d.HIS1, his1△/ his1△, arg4△/ arg4△, leu2△/ leu2△, URA3/ura3△::imm^434^ IRO1/iro1△::imm^434^* | This study |
| *hap2△/△* | SN152 | *hap2△::C.m.LEU2/hap2△::C.d.HIS1, his1△/ his1△, arg4△/ arg4△, leu2△/ leu2△, URA3/ura3△::imm^434^ IRO1/iro1△::imm^434^* | This study |
| *hap2△/△+HAP2* | *hap2△/△* | *hap2△::HAP2::C.d.ARG4/hap2△::C.d.HIS1, his1△/ his1△, arg4△/ arg4△, leu2△/ leu2△, URA3/ura3△::imm^434^ IRO1/iro1△::imm^434^* | This study |
| *rpn4△/△* | SN152 | *rpn4△::C.m.LEU2/rpn4△::C.d.HIS1, his1△/ his1△, arg4△/ arg4△, leu2△/ leu2△, URA3/ura3△::imm^434^ IRO1/iro1△::imm^434^* | This study |
| *try5△/△* | SN152 | *try5△::C.m.LEU2/ try5△::C.d.HIS1, his1△/ his1△, arg4△/ arg4△, leu2△/ leu2△, URA3/ura3△::imm^434^ IRO1/iro1△::imm^434^* | This study |
| *bas1△/△* | SN152 | *bas1△::C.m.LEU2/ bas1△::C.d.HIS1, his1△/ his1△, arg4△/ arg4△, leu2△/ leu2△, URA3/ura3△::imm^434^ IRO1/iro1△::imm^434^* | This study |
| *ctf1△/△* | SN152 | *ctf1△::C.m.LEU2/ ctf1△::C.d.HIS1, his1△/ his1△, arg4△/ arg4△, leu2△/ leu2△, URA3/ura3△::imm^434^ IRO1/iro1△::imm^434^* | This study |
| *sfl1△/△* | SN152 | *sfl1△::C.m.LEU2/ sfl1△::C.d.HIS1, his1△/ his1△, arg4△/ arg4△, leu2△/ leu2△, URA3/ura3△::imm^434^ IRO1/iro1△::imm^434^* | This study |
| *leu3△/△* | SN152 | *leu3△::C.m.LEU2/ leu3△::C.d.HIS1, his1△/ his1△, arg4△/ arg4△, leu2△/ leu2△, URA3/ura3△::imm^434^ IRO1/iro1△::imm^434^* | This study |
| *rme1△/△* | SN152 | *rme1△::C.m.LEU2/ rme1△::C.d.HIS1, his1△/ his1△, arg4△/ arg4△, leu2△/ leu2△, URA3/ura3△::imm^434^ IRO1/iro1△::imm^434^* | This study |
| *tye7△/△* | SN152 | *tye7△::C.m.LEU2/ tye7△::C.d.HIS1, his1△/ his1△, arg4△/ arg4△, leu2△/ leu2△, URA3/ura3△::imm^434^ IRO1/iro1△::imm^434^* | This study |
| *orf19.5651△/△* | SN152 | *orf19.5651△::C.m.LEU2/* *orf19.5651△::C.d.HIS1, his1△/ his1△, arg4△/ arg4△, leu2△/ leu2△, URA3/ura3△::imm^434^ IRO1/iro1△::imm^434^* | This study |
| *cup9△/△* | SN152 | *cup9△::C.m.LEU2/ cup9△::C.d.HIS1, his1△/ his1△, arg4△/ arg4△, leu2△/ leu2△, URA3/ura3△::imm^434^ IRO1/iro1△::imm^434^* | This study |
| *yox1△/△* | SN152 | *yox1△::C.m.LEU2/ yox1△::C.d.HIS1, his1△/ his1△, arg4△/ arg4△, leu2△/ leu2△, URA3/ura3△::imm^434^ IRO1/iro1△::imm^434^* | This study |
| *zcf39△/△* | SN152 | *zcf39△::C.m.LEU2/ zcf39△::C.d.HIS1, his1△/ his1△, arg4△/ arg4△, leu2△/ leu2△, URA3/ura3△::imm^434^ IRO1/iro1△::imm^434^* | This study |
| *war1△/△* | SN152 | *war1△::C.m.LEU2/ war1△::C.d.HIS1, his1△/ his1△, arg4△/ arg4△, leu2△/ leu2△, URA3/ura3△::imm^434^ IRO1/iro1△::imm^434^* | This study |
| *cat8△/△* | SN152 | *cat8△::C.m.LEU2/ cat8△::C.d.HIS1, his1△/ his1△, arg4△/ arg4△, leu2△/ leu2△, URA3/ura3△::imm^434^ IRO1/iro1△::imm^434^* | This study |
| *orf19.1496△/△* | SN152 | *orf19.1496△::C.m.LEU2/ orf19.1496△::C.d.HIS1, his1△/ his1△, arg4△/ arg4△, leu2△/ leu2△, URA3/ura3△::imm^434^ IRO1/iro1△::imm^434^* | This study |
| *hms1△/△* | SN152 | *cat8△::C.m.LEU2/ cat8△::C.d.HIS1, his1△/ his1△, arg4△/ arg4△, leu2△/ leu2△, URA3/ura3△::imm^434^ IRO1/iro1△::imm^434^* | This study |
| *pho4△/△* | SN152 | *pho4△::C.m.LEU2/ pho4△::C.d.HIS1, his1△/ his1△, arg4△/ arg4△, leu2△/ leu2△, URA3/ura3△::imm^434^ IRO1/iro1△::imm^434^* | This study |
| *zcf13△/△* | SN152 | *zcf13△::C.m.LEU2/ zcf13△::C.d.HIS1, his1△/ his1△, arg4△/ arg4△, leu2△/ leu2△, URA3/ura3△::imm^434^ IRO1/iro1△::imm^434^* | This study |
| *sef1△/△* | SN152 | *sef1△::C.m.LEU2/ sef1△::C.d.HIS1, his1△/ his1△, arg4△/ arg4△, leu2△/ leu2△, URA3/ura3△::imm^434^ IRO1/iro1△::imm^434^* | This study |
| *gln3△/△* | SN152 | *gln3△::C.m.LEU2/ gln3△::C.d.HIS1, his1△/ his1△, arg4△/ arg4△, leu2△/ leu2△, URA3/ura3△::imm^434^ IRO1/iro1△::imm^434^* | This study |
| *cta7△/△* | SN152 | *cta7△::C.m.LEU2/ cta7△::C.d.HIS1, his1△/ his1△, arg4△/ arg4△, leu2△/ leu2△, URA3/ura3△::imm^434^ IRO1/iro1△::imm^434^* | This study |
| *stp1△/△* | SN152 | *stp1△::C.m.LEU2/ stp1△::C.d.HIS1, his1△/ his1△, arg4△/ arg4△, leu2△/ leu2△, URA3/ura3△::imm^434^ IRO1/iro1△::imm^434^* | This study |
| *zcf24△/△* | SN152 | *zcf24△::C.m.LEU2/ zcf24△::C.d.HIS1, his1△/ his1△, arg4△/ arg4△, leu2△/ leu2△, URA3/ura3△::imm^434^ IRO1/iro1△::imm^434^* | This study |
| *cup2△/△* | SN152 | *cup2△::C.m.LEU2/ cup2△::C.d.HIS1, his1△/ his1△, arg4△/ arg4△, leu2△/ leu2△, URA3/ura3△::imm^434^ IRO1/iro1△::imm^434^* | This study |
| *mbp1△/△* | SN152 | *mbp1△::C.m.LEU2/ mbp1△::C.d.HIS1, his1△/ his1△, arg4△/ arg4△, leu2△/ leu2△, URA3/ura3△::imm^434^ IRO1/iro1△::imm^434^* | This study |
| *zcf32△/△* | SN152 | *zcf32△::C.m.LEU2/ zcf32△::C.d.HIS1, his1△/ his1△, arg4△/ arg4△, leu2△/ leu2△, URA3/ura3△::imm^434^ IRO1/iro1△::imm^434^* | This study |
| *orf19.6874△/△* | SN152 | *orf19.6874△::C.m.LEU2/ orf19.6874△::C.d.HIS1, his1△/ his1△, arg4△/ arg4△, leu2△/ leu2△, URA3/ura3△::imm^434^ IRO1/iro1△::imm^434^* | This study |
| *uga33△/△* | SN152 | *uga33△::C.m.LEU2/ uga33△::C.d.HIS1, his1△/ his1△, arg4△/ arg4△, leu2△/ leu2△, URA3/ura3△::imm^434^ IRO1/iro1△::imm^434^* | This study |
| *orf19.217△/△* | SN152 | *orf19.217△::C.m.LEU2/ orf19.217△::C.d.HIS1, his1△/ his1△, arg4△/ arg4△, leu2△/ leu2△, URA3/ura3△::imm^434^ IRO1/iro1△::imm^434^* | This study |
| *zcf2△/△* | SN152 | *zcf2△::C.m.LEU2/ zcf2△::C.d.HIS1, his1△/ his1△, arg4△/ arg4△, leu2△/ leu2△, URA3/ura3△::imm^434^ IRO1/iro1△::imm^434^* | This study |
| *orf19.1274△/△* | SN152 | *orf19.1274△::C.m.LEU2/ orf19.1274△::C.d.HIS1, his1△/ his1△, arg4△/ arg4△, leu2△/ leu2△, URA3/ura3△::imm^434^ IRO1/iro1△::imm^434^* | This study |
| *stb5△/△* | SN152 | *stb5△::C.m.LEU2/ stb5△::C.d.HIS1, his1△/ his1△, arg4△/ arg4△, leu2△/ leu2△, URA3/ura3△::imm^434^ IRO1/iro1△::imm^434^* | This study |
| *zcf19△/△* | SN152 | *zcf19△::C.m.LEU2/ zcf19△::C.d.HIS1, his1△/ his1△, arg4△/ arg4△, leu2△/ leu2△, URA3/ura3△::imm^434^ IRO1/iro1△::imm^434^* | This study |
| *zcf22△/△* | SN152 | *zcf22△::C.m.LEU2/ zcf22△::C.d.HIS1, his1△/ his1△, arg4△/ arg4△, leu2△/ leu2△, URA3/ura3△::imm^434^ IRO1/iro1△::imm^434^* | This study |
| *zcf23△/△* | SN152 | *zcf23△::C.m.LEU2/ zcf23△::C.d.HIS1, his1△/ his1△, arg4△/ arg4△, leu2△/ leu2△, URA3/ura3△::imm^434^ IRO1/iro1△::imm^434^* | This study |
| *orf19.4972△/△* | SN152 | *orf19.4972△::C.m.LEU2/ orf19.4972△::C.d.HIS1, his1△/ his1△, arg4△/ arg4△, leu2△/ leu2△, URA3/ura3△::imm^434^ IRO1/iro1△::imm^434^* | This study |
| *orf19.5849△/△* | SN152 | *orf19.5849△::C.m.LEU2/ orf19.5849△::C.d.HIS1, his1△/ his1△, arg4△/ arg4△, leu2△/ leu2△, URA3/ura3△::imm^434^ IRO1/iro1△::imm^434^* | This study |
| *zcf31△/△* | SN152 | *zcf31△::C.m.LEU2/ zcf31△::C.d.HIS1, his1△/ his1△, arg4△/ arg4△, leu2△/ leu2△, URA3/ura3△::imm^434^ IRO1/iro1△::imm^434^* | This study |
| *try6△/△* | SN152 | *try6△::C.m.LEU2/ try6△::C.d.HIS1, his1△/ his1△, arg4△/ arg4△, leu2△/ leu2△, URA3/ura3△::imm^434^ IRO1/iro1△::imm^434^* | This study |
| *mac1△/△* | SN152 | *mac1△::C.m.LEU2/ mac1△::C.d.HIS1, his1△/ his1△, arg4△/ arg4△, leu2△/ leu2△, URA3/ura3△::imm^434^ IRO1/iro1△::imm^434^* | This study |
| *asg1△/△* | SN152 | *asg1△::C.m.LEU2/ asg1△::C.d.HIS1, his1△/ his1△, arg4△/ arg4△, leu2△/ leu2△, URA3/ura3△::imm^434^ IRO1/iro1△::imm^434^* | This study |
| *upc2△/△* | SN152 | *upc2△::C.m.LEU2/ upc2△::C.d.HIS1, his1△/ his1△, arg4△/ arg4△, leu2△/ leu2△, URA3/ura3△::imm^434^ IRO1/iro1△::imm^434^* | This study |
| *zcf3△/△* | SN152 | *zcf3△::C.m.LEU2/ zcf3△::C.d.HIS1, his1△/ his1△, arg4△/ arg4△, leu2△/ leu2△, URA3/ura3△::imm^434^ IRO1/iro1△::imm^434^* | This study |
| *orf19.5026△/△* | SN152 | *orf19.5026△::C.m.LEU2/ orf19.5026△::C.d.HIS1, his1△/ his1△, arg4△/ arg4△, leu2△/ leu2△, URA3/ura3△::imm^434^ IRO1/iro1△::imm^434^* | This study |
| *zcf29△/△* | SN152 | *zcf29△::C.m.LEU2/ zcf29△::C.d.HIS1, his1△/ his1△, arg4△/ arg4△, leu2△/ leu2△, URA3/ura3△::imm^434^ IRO1/iro1△::imm^434^* | This study |
| *opi1△/△* | SN152 | *opi1△::C.m.LEU2/ opi1△::C.d.HIS1, his1△/ his1△, arg4△/ arg4△, leu2△/ leu2△, URA3/ura3△::imm^434^ IRO1/iro1△::imm^434^* | This study |
| *zcf1△/△* | SN152 | *zcf1△::C.m.LEU2/ zcf1△::C.d.HIS1, his1△/ his1△, arg4△/ arg4△, leu2△/ leu2△, URA3/ura3△::imm^434^ IRO1/iro1△::imm^434^* | This study |

**Tables S2. Primes used in this study.**

| **Primer**  **name** | **Primer sequence (5'-3')** |
| --- | --- |
| W1 | GGCGAATTGGAGCTCCACCGCGGTGGCGGCCGCTCTAGAACTAGTGGATCGTTTAAACTTGGTAGATTTACAACTGAAGCCG |
| W2 | GAAAGCTTGAATTGATTGATTGATTGAAAAGGGGAGTATTTCTGGAGTGAA |
| W3 | TTCACTCCAGAAATACTCCCCTTTTCAATCAATCAATCAATTCAAGCTTTC |
| W4 | CGATACATTTGCGGTACAGAAATGTTTAACTTGTCGGAAGAGAAAGAAGA |
| W5 | CTAGTGGTGGTGGTATCAGTAGATACATTTCTGTACCGCAAATGTATCG |
| W6 | TAAATGCTAACTACTGTATATACTGAGTAATGAAAGCAGTCAAAGGGCTC |
| W7 | GAGCCCTTTGACTGCTTTCATTACTCAGTATATACAGTAGTTAGCATTTA |
| W8 | GTCGACGGTATCGATAAGCTTGATATCGAATTCCTGCAGCCCGGGGGATCGTTTAAACTCGAAAACGATGTTTGCACCACCG |
| *CDR1*-F | ATGTCAGATTCTAAGATG |
| *CDR1*-R | CAGGATCTGATTCAAACA |
| *MDR1*-F | GCCCATTGGTTTTCAGTCCG |
| *MDR1*-R | CACCACCAGTGGCCAAACAA |
| *ERG3*-F | TGCCACTACTGCCATTCCAG |
| *ERG3*-R | GGCCAGTGTAACCATCTATG |
| *ERG11*-F | GGGTTGCCAATGTTATGAAAACTCA |
| *ERG11*-R | ATTTTCTTTTGAGCAGCATCACGTC |
| *SOD1-*F | CAACATGGTGCTCCAGAAGA |
| *SOD1-*R | TCTAGCACCAGCATGACCAG |
| *SOD2-*F | TCAATTGAACAAGCCGTTGAAGCCAAA |
| *SOD2-*R | ACCACCTTGAGAGACAGGAGCCA |
| *SOD3-*F | CGCTATTGACGCACTTGAAA |
| *SOD3-*R | TTGTTTACCCAAAGCGGAAC |
| *SOD4-*F | AGGCAAGGCACCATTAGTTG |
| *SOD4-*R | TTTTGGATTCTGGAACTGGC |
| *SOD5-*F | TTCATGAAAAACCAGTGCCA |
| *SOD5-*R | TCACCAACTTCATGAGCAGC |
| *SOD6-*F | ACAGGTCAAAGGAAACGTGG |
| *SOD6-*R | CATCGCAAACTGGACTAGCA |
| *CAT3-*F | GTTTCGTGACGCAAGAACTG |
| *CAT3-*R | CTCCAGTGGACTAGCATTGGT |
| *ACT*-F | GTGGTACTACCATGTTCCCAGG |
| *ACT*-R | GATAGAACCACCAATCCAGACAGAG |

F, forward; R, reverse.

**Table S3. MIC (μg/mL) values of 48 *Candida albicans* transcription factor-deficient strains to fluconazole and miconazole.**

| **Strain** | **Systematic name** | **Gene name** | **Function** | **MIC (μg/mL)** | | | |
| --- | --- | --- | --- | --- | --- | --- | --- |
|  |  |  |  | **FLC (24 h)** | **MCZ (24 h)** | **FLC (48 h)** | **MCZ (48 h)** |
| SN250 | - | - | - | ＞64 | 16 | ＞64 | 16 |
| No.01 | orf19.1228 | *HAP2* | Regulation of low-iron induction of *FRP1* | <0.125 | <0.125 | 0.25 | <0.125 |
| No.02 | orf19.1253 | *PHO4* | Phosphate acquisition and resistance to stress | 0.25 | 8 | 0.5 | 8 |
| No.03 | orf19.1069 | *RPN4* | Proteasome genes regulation | 0.25 | 8 | 0.25 | 8 |
| No.04 | orf19.454 | *SFL1* | Negative regulation of morphogenesis | 0.25 | 8 | 32 | 8 |
| No.05 | orf19.2646 | *ZCF13* | *In vivo* colonization regulation | 1 | 16 | 32 | 16 |
| No.06 | orf19.391 | *UPC2* | Regulation of ergosterol biosynthetic | 1 | <0.125 | 1 | 8 |
| No.07 | orf19.3809 | *BAS1* | Purine biosynthetic genes regulation | 4 | <0.125 | 4 | 16 |
| No.08 | orf19.4225 | *LEU3* | Regulation of amino acid biosynthesis genes | 4 | <0.125 | 32 | 8 |
| No.09 | orf19.5924 | *ZCF31* | Unknown | 4 | 8 | 32 | 16 |
| No.10 | orf19.4450 | *ZCF23* | Unknown | 8 | 8 | 32 | 8 |
| No.11 | orf19.5001 | *CUP2* | Regulation of resistance to copper | 8 | 8 | 32 | 8 |
| No.12 | orf19.6874 | *C2_05640W* | Regulation of filamentous growth | 8 | 8 | 32 | 16 |
| No.13 | orf19.5026 | *C1_13880C* | Unknown | 8 | 16 | 32 | 8 |
| No.14 | orf19.7068 | *MAC1* | Regulation of copper transporter | 8 | 16 | 32 | 8 |
| No.15 | orf19.921 | *HMS1* | Morphogenesis regulation | 8 | 16 | 32 | 16 |
| No.16 | orf19.5849 | *CWT1* | Negative regulation of nitrosative stress response | 8 | 16 | 32 | 16 |
| No.17 | orf19.1499 | *CTF1* | Activation of fatty acid degradation genes | 8 | 16 | 32 | 16 |
| No.18 | orf19.1168 | *ZCF3* | Required for filamentous growth | 16 | 2 | 128 | 16 |
| No.19 | orf19.3308 | *STB5* | *YOR1* overexpression induction | 32 | 16 | 32 | 16 |
| No.20 | orf19.3434 | *TRY5* | Yeast form adherence regulation | 32 | 4 | 32 | 16 |
| No.21 | orf19.3753 | *SEF1* | Iron uptake regulation | 32 | 4 | 32 | 8 |
| No.22 | orf19.3876 | *ZCF19* | Unknown | 32 | 16 | 32 | 16 |
| No.23 | orf19.3912 | *GLN3* | Regulation of filamentous growth | 32 | 16 | 32 | 16 |
| No.24 | orf19.4251 | *ZCF22* | Unknown | 32 | 8 | 32 | 8 |
| No.25 | orf19.4288 | *CTA7* | Activation of 1-hybrid assay in *S. cerevisiae* | 32 | 8 | 32 | 8 |
| No.26 | orf19.4438 | *RME1* | Asexual sporulation regulation | 32 | 8 | 32 | 8 |
| No.27 | orf19.4524 | *ZCF24* | Unknown | 32 | 8 | 32 | 8 |
| No.28 | orf19.4941 | *TYE7* | Regulation of glycolysis and biofilm formation | 32 | 8 | 32 | 8 |
| No.29 | orf19.4972 | *C1_13440C* | Regulation of white-opaque switching and filamentous growth | 32 | 8 | 32 | 8 |
| No.30 | orf19.5097 | *CAT8* | Regulation of filamentous growth | 32 | 8 | 32 | 8 |
| No.31 | orf19.5133 | *ZCF29* | Regulation of sensitivity to caffeine, menadione, beauvericin and fenpropimorph | 32 | 8 | 32 | 8 |
| No.32 | orf19.5917 | *STP1* | Regulation of protein catabolism for nitrogen source | 32 | 8 | 32 | 8 |
| No.33 | orf19.5940 | *ZCF32* | Regulation of biofilm formation | 32 | 8 | 32 | 8 |
| No.34 | orf19.5651 | *C4_00260W* | Unknown | 32 | 8 | 32 | 16 |
| No.35 | orf19.5855 | *MBP1* | Regulation of G1/S cell-cycle progression | 32 | 8 | 32 | 16 |
| No.36 | orf19.6824 | *TRY6* | Regulation of yeast form adherence | 32 | 8 | 32 | 16 |
| No.37 | orf19.7017 | *YOX1* | Unknown | 32 | 8 | 32 | 16 |
| No.38 | orf19.6514 | *CUP9* | *SOK1* expression repression in response to farnesol inhibition | 32 | 16 | 32 | 8 |
| No.39 | orf19.7317 | *UGA33* | Regulation of γ-aminobutyrate metabolism | 32 | 16 | 32 | 16 |
| No.40 | orf19.1496 | *C2_01870C* | Unknown | ＞64 | 0.5 | ＞64 | 0.5 |
| No.41 | orf19.1543 | *OPI1* | Regulation of filamentous growth | ＞64 | 8 | ＞64 | 8 |
| No.42 | orf19.7583 | *ZCF39* | Regulation of filamentation growth and yeast cell adherence | ＞64 | 8 | ＞64 | 16 |
| No.43 | orf19.166 | *ASG1* | Regulation of growth on non-fermentative carbon sources | ＞64 | 8 | ＞64 | 16 |
| No.44 | orf19.217 | *C2_08890W* | Regulation of iron homeostasis and morphogenesis | ＞64 | 8 | ＞64 | 16 |
| No.45 | orf19.255 | *ZCF1* | Transcription regulation during hypha formation | ＞64 | 8 | ＞64 | 16 |
| No.46 | orf19.431 | *ZCF2* | Regulation of sulfite tolerance | ＞64 | 8 | ＞64 | 16 |
| No.47 | orf19.1035 | *WAR1* | Regulation of resistance to weak organic acids and yeast cell adherence | ＞64 | 8 | ＞64 | 16 |
| No.48 | orf19.1274 | *C4_05870C* | Unknown | ＞64 | 8 | ＞64 | 16 |

Abbreviation: FLC, fluconazole; MCZ, miconazole.


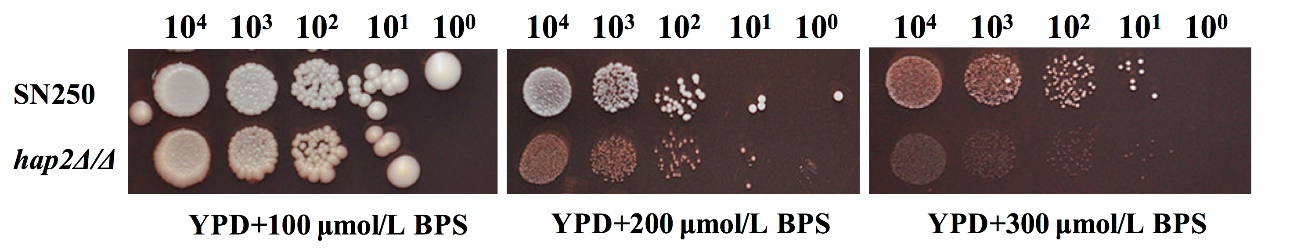


**Figure S1.** Effect of *hap2Δ/Δ* mutation on *Candida albicans* growth. *Candida albicans* control strain (SN250 and *hap2Δ/Δ* mutant were grown in iron-limited medium with 100 μmol/L, 200 μmol/L or 300 μmol/L BPS. Ten-fold serial dilutions were spotted to the plates and incubated at 30 ˚C for 48 h.
